# INCORPORATING NEW MEASURES OF NEIGHBORHOOD SUPPORTS FOR HEALTHY AGING INTO EXISTING COHORTS

**DOI:** 10.1093/geroni/igad104.0021

**Published:** 2023-12-21

**Authors:** Jana Hirsch, Yvonne Michael

**Affiliations:** Dornsife School of Public Health Drexel University, Philadelphia, Pennsylvania, United States; Drexel University, Philadelphia, Pennsylvania, United States

## Abstract

Longstanding cohort studies have a wealth of well-characterized sociodemographic, phenotypic, behavioral, genetic, health, and neighborhood longitudinal data, often spanning decades. In addition to original aims, we have an obligation to leverage the current cohort studies to better understand the aging process and neighborhood supports for healthy aging. We used a multi-pronged approach to design and add aging-relevant neighborhood measures to the Multi-Ethnic Study of Atherosclerosis (MESA) during Exam 7 (ongoing since 2022), when the entire cohort had aged to >67 years. In addition to continuing survey measures from historic visits on food environments, safety, social cohesion, trust, and physical activity resources we added novel metrics to the MESA Neighborhood Ancillary questionnaire. These include across environmental domains relevant for physical function (e.g. curb cuts, availability of places to rest, lighting, crosswalk speeds), cognitive function (e.g. places to learn new things, signage), and ability to age in place (e.g. neighborhood changes related to cost or pressures). To complement existing Geographic Information Systems (GIS) data in MESA, we collected and developed new GIS measures similarly related to these three domains: physical function (e.g. senior center density, physical therapist density), cognitive function (e.g. destinations to enrich cognitive function, places to socialize, new greenness measures), and ability to age in place (e.g. age isolation, age-friendly policies). Our ability to leverage decades of data on well-characterized cohorts by complementing existing data with new, neighborhood supports for healthy aging opens novel research opportunities to understand health across the lifecourse.

